# Result of Observations and Investigations Made in the United States Marine Hospital, Chicago, during the Quarter, Ending July 1, 1852

**Published:** 1852-09

**Authors:** 


					﻿Results of Observati ns and Investigations made in the U. S. Mirine Hospital.
Chicago, during the Quarter, ending July 1, 1852.
The U. S. Marine Hospital at Chicago was organized and opened
for the reception of patients on the 1st of April. Since that time
it has supplied and is still furnishing to the writer, acting as he is
in the capacity of surgeon and physician to the institution, an op-
portunity for investigation and the observation of medical and sur-
gical facts, which will be made the basis of a scries of Quarterly
Reports for the editorial department of our Journal.
The number of cases admitted during the quarter was less than
might have been anticipated at a port of so much commercial im-
portance as Chicago. This resulted, in part, from the fact that at
the time of organizing the hospital, and for a month or two after,
it was not generally known among sailors and boatmen that such
an institution had been opened; and, in part, from the almost entire
exemption of all classes from sickness during the months of April.
May and June, of this year.
The wards have high ceilings and are well ventilated, and are so
separated as to admit of the destribution of patients to any desirable
extent.
Adjacent to the wards there are bath rooms, having all the con-
veniences for furnishing at any time, and as soon as ordered, cold,
warm and shower baths. Among the general rules adapted for
the treatment of all cases, are the following:—"All patients, on
being admitted to the Hospital, are taken at once to the bath room,
thoroughly cleansed, supplied with clean underclothes, and then
provided with a bed in some ward occupied by patients whose
diseases .are the same or similar in character.
After this the daily prescription embraces full directions with
regard to diet and regimen as well as the administration of medi-
cine.
Having thus given a general view of the treatment adopted in
nearly all the cases presented, we will now pass to the considera-
tion of what we believe to be some of the most interesting and
important facts observed during the progress and treatment of
special diseases.
The following table, showing the nature of the diseases, and the
average time under treatment, of the several cases admitted during
the quarter, will serve as the basis for our remarks:—
o 13-	5 io 5 is 5	.5	£>	5-5
“ .—7 ■ , "2 Si'S	x "2 i’e-
i- a g ■- a	a p ' 2 a '2 3 15 ” op Z z o’ J o 'z ' oz
± 5	~ r Ut ~ w-	- U;'ui 2Uh — 'a Ur ; '.-.h
~	— ’>	>	- tZ	’>	!'>	= i -
Andi.	4	7	4 15	1	8	|	j 1| 14	18
Maz	7	8	40	2 l(i 2	8	1 15 1 2	8 I	1	44	1 10 K 11
Jiine.	9	0	1	2 82	2 18 : 1	7	1 50	2	8	11
Tn-al.	20	7	27! 3 12	2	8 I 3 IS	13	2_ 104	2 jf_____9 2^ 1°‘	04
				

